# Intracellular persister: A stealth agent recalcitrant to antibiotics

**DOI:** 10.3389/fcimb.2023.1141868

**Published:** 2023-03-31

**Authors:** Nicolas Personnic, Patricia Doublet, Sophie Jarraud

**Affiliations:** ^1^ CIRI, Centre International de Recherche en Infectiologie, CNRS UMR 5308, INSERM U1111, Ecole Normale Supérieure de Lyon, Université Claude Bernard Lyon 1, Lyon, France; ^2^ Group Persistence and Single-Cell Dynamics of Respiratory Pathogens, Lyon, France; ^3^ Group Legionella Pathogenesis, Lyon, France; ^4^ National Reference Centre for Legionella, Institute of Infectious Agents, Hospices Civils de Lyon, Lyon, France

**Keywords:** persisters, antibiotic, heterogeneity, infection, virulence

## Abstract

The bulk of bacteria transiently evading appropriate antibiotic regimes and recovered from non-resolutive infections are commonly refer to as persisters. In this mini-review, we discuss how antibiotic persisters stem from the interplay between the pathogen and the cellular defenses mechanisms and its underlying heterogeneity.

## Introduction

In clinics, many pathogens are hard to eradicate even in the absence of genetically detectable anti-microbial resistance (AMR) mechanisms, and despite proven antibiotic susceptibilities in antimicrobial sensitivity testing (AST) (2019). The bulk of bacteria transiently evading appropriate antibiotic regimes and recovered from non-resolutive diseases are commonly refer to as persisters (**Box 1**). The formation of persisters has been experimentally documented for major bacterial pathogens including *Staphylococcus aureus* (Huemer et al., 2021), *Mycobacterium tuberculosis* (Manina et al., 2015), *Escherichia coli* (Kerkez et al., 2021), *Salmonella enterica* (Helaine et al., 2014), *Pseudomonas* spp (Mulcahy et al., 2010)., *Listeria monocytogenes* (Kortebi et al., 2017), *Legionella pneumophila* (Personnic et al., 2019)*, Burkholderia pseudomallei* (Ross et al., 2019), and *Yersinia pseudo-tuberculosis* (Raneses et al., 2020).

**Box 1**

Antibiotic persister: Two visions regularly collide with each other to define the persister and can explain many puzzling contradictions that have appeared in the literatures. On the one hand, the persisters correspond to a fraction of the antibiotic susceptible bacteria that colonize alternative tissues, cellular or intra-cellular compartments (biogeography hypothesis). This results in local alteration of the antibiotic pharmaco-dynamics that “accidentally” reduces the efficiency of the antimicrobial chemotherapy (Bakkeren et al., 2019; Santucci et al., 2021; Sharma et al., 2021; Van Bambeke et al., 2006). Alternatively, bacteria may encounter microenvironments with restrictive conditions (nutritional deprivation, physical and chemical stress, etc.) that favor the development of phenotypic traits that increase the bacterial recalcitrance to the antibiotic (e.g., slow to no growth, reduced metabolism, increased stress responses, etc.) (Claudi et al., 2014; Demarre et al., 2019; Helaine et al., 2014; Huemer et al., 2021; Kortebi et al., 2017; Manina et al., 2015; Peyrusson et al., 2020; Raneses et al., 2020; Rowe et al., 2020; Vulin et al., 2018). On the other hand, bacterial persisters would be the expression of a genetically encoded strategy through which some isogenic individuals, among an otherwise clonal and antibiotic susceptible pathogen population, nested in the very same niche, transiently adopt a physiology recalcitrant to the antibiotic treatment (phenotypic heterogeneity hypothesis) (Balaban et al., 2004; Conlon et al., 2016; Manuse et al., 2021; Mishra et al., 2019; Personnic et al., 2019; Personnic et al., 2021; Wakamoto et al., 2013). Obviously, the underlying molecular mechanisms are highly diverse and the single-cell level of analysis is essential to unveil and to untangle them.

Many mechanisms leading to the formation of persisters has been deciphered essentially using culture in broth (Balaban et al., 2004; Wakamoto et al., 2013; Conlon et al., 2016; Manuse et al., 2021 Molina-Quiroz et al., 2018; Moyed and Bertrand, 1983; Personnic et al., 2021; Ross et al., 2018). To which extent the mechanisms inferred can apply “*in cellulo*” or “*in vivo*” remains largely unexplored. The persisters comprise both metabolically active individuals, and/or individuals with dormant-like features (*i.e.*, growth arrest, low to no metabolism). Recent work proposes that the level of dormancy of the persisters is function of the level of stress undergone, the most extreme expression being the viable but non-cultivable bacteria [VBNC (Peyrusson et al., 2022), reviewed in (Ayrapetyan et al., 2018)].

This mini-review is centered on the works performed in condition of infections. We decided to term persister, any intracellular bacterial individual evading the combined action of the host and the antibiotic treatment and that results from the heterogeneity in the biological processes and treatment efficacy. To get more insight on the definitions, and controversies, about the phenomenon of antibiotic recalcitrance in broth or during the infection (*i.e.*, antibiotic persistence, antibiotic hetero-tolerance and antibiotic tolerance), we invite the reader to consult the many excellent review on the topic (Ayrapetyan et al., 2018; Balaban et al., 2013; Balaban et al., 2019; Brauner et al., 2016; Grant and Hung, 2013; Gollan et al., 2019; Harms et al., 2016; Lewis, 2010; Levin et al., 2014; Nathan, 2012; Ronneau et al., 2021):

During an infection, the inherent heterogeneity in the biological processes and treatment efficacy contributes decisively to the formation of antibiotic persisters. It encompasses (i) the divergent antibiotic penetration and activation at the tissue, cellular and subcellular level (Bakkeren et al., 2019; Santucci et al., 2021; Sharma et al., 2021); (ii) the disparate host-pathogen interactions within structured tissue and/or lesions that undermine the bactericidal activities of the antibiotics and the host defenses (Bumann, 2015; Bumann and Cunrath, 2017; Claudi et al., 2014; Li et al., 2021; Sharma et al., 2021); and (iii) the transient and reversible adoption by some individual bacteria of physiological traits rendering them recalcitrant to the antibiotics (Beam et al., 2021; Claudi et al., 2014; Demarre et al., 2019; Helaine et al., 2014; Huemer et al., 2021; Kerkez et al., 2021; Kortebi et al., 2017; Leimer et al., 2016; Manina et al., 2015; Mishra et al., 2019; Personnic et al., 2019; Raneses et al., 2020; Rowe et al., 2020; Vulin et al., 2018).

Recent works indicate that, at the core of this phenomenon, is the interaction between the pathogen and a cellular host. Actually, for various bacterial species, persisters frequently emerge intra-cellularly notably in macrophages that normally contribute to the first line of defense to control the pathogen burden. Intracellular persisters have been also documented in protozoa indicating that the ability to produce antibiotic persisters also applies to evolutionarily distant host cells and predates the emergence of metazoans (Personnic et al., 2019).

In this mini-review, we discuss how the bacteria-cell interplay drives both the formation and the survival of the antibiotic persisters, and to which extent it is governed by the heterogeneity in the biological systems.

## Persisters formation: A response to the cellular defenses

**Figure 1:**
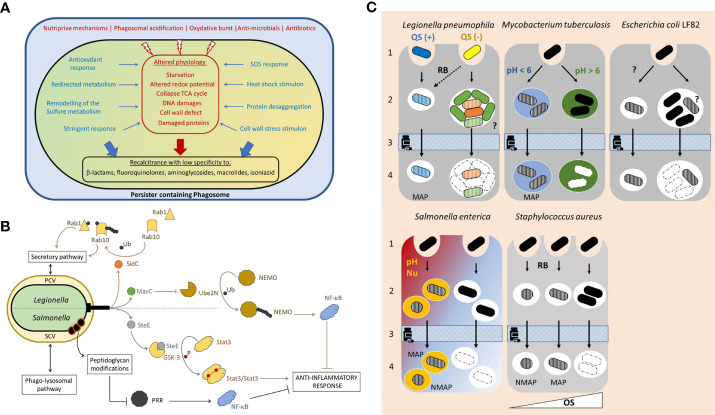
**(A)** Formation of an intravacuolar persister. Quickly after uptake, the phagocytic vacuole maturates along the endo-lysosomal pathway. Within the phagosome various stressors contribute to the pathogen clearance. Both the alteration of the bacterial physiology (depicted in red) and the bacterial adaptative responses, to counter the macrophage-derived stress (“defensive mode” depicted in blue), favor (large blue and red arrows) the formation of a specialized subpopulation of survivors with high recalcitrance to antibiotics termed intracellular persisters (depicted in black). **(B)** The intravacuolar persisters undermine the host defense. In addition to their defensive mode, intracellular *Salmonella* and *Legionella* persisters can execute an offensive virulence program that translocated effectors *via* a type 3 or type 4 secretion system, respectively. Contrary to the *Salmonella* virulence factor SteE produced both by the persisters and the coexisting antibiotic susceptible individuals, the *Legionella* intracellular persisters specifically produced SidC and MavC. The figure depicts the mode of action those 3 effectors, the modes of action of which are detailed in the main text. Pattern Recognition Receptors (PRR) recognize molecules frequently found in pathogen and trigger pro-inflammatory response enhancing the bactericidal activities. Persisters may tackle immune recognition by modifying their peptidoglycan. PCV, persister containing vacuole; SCV, *Salmonella* containing vacuole; Ub, ubiquitination; P, phosphorylation. **(C)** Persisters formation stems from heterogeneity in biological processes. The underlying mechanisms remain largely unexplored. For *L. pneumophila:* alternative activation of the quorum sensing (blue and yellow bacteria) and intravacuolar heterogenous differentiation of the proliferative subset (green and orange bacteria). For *M. tuberculosis*: phagosome to phagosome pH variations depicted by different colors. For *E. coli* LF82: unknown. Persisters formation may result from heterogeneous stress exposure or response. For *S. enterica*: Alternative macrophage polarization (proinflammatory in red, anti-inflammatory in blue) possibly associated to distinct phagosomal features (pH and nutrients). For *S. aureus*: alternative oxidative stress pressure generating distinct persisters (*i.e.*, with distinct dormancy’s depth depicted by the bacterial shape). QS, quorum sensing; RB, respiratory burst; Nu, nutrient limitation; OS, oxidative stress (strength depicted with a white triangle); MAP, metabolically active persisters; NMAP, non-metabolically active persisters; filled bacterium, antibiotic susceptible bacterium; hatched bacterium, antibiotic persister; dashed bacteria, dead individual;?, unknown mechanism. 1, uptake; 2 persister formation or intracellular proliferation of antibiotic susceptible bacteria; 3, antibiotic treatment; 4, Persister survival.

Phagocytosis is an evolutionarily ancient and conserved component of defense against pathogen invasion. Within the phagocytic vacuole, a mosaic of bacterial adaptative responses to the host cell-derived stress can take the form of a specialized subpopulation of survivors that are highly tolerant to one or various antibiotics (**Figure 1A**). Such intracellular persisters are traceable using fluorescence-based high-throughput single-cell technologies (**Box 2**).

**Box 2**

Tracking the persisters: Recent advances in fluorescence-based single-cell technologies, specifically various fluorescent probes that confer the persisters with specific spectral properties, rendered possible quantitative tracking and analyses the pre-existing pool of persisters, *in situ*, and without the need of an artificial clearance of the other subpopulations by using antibiotics at high concentrations. Single-cell fluorescent growth rate reporters are recognized as a method of choice to track the persisters on the bases of their lack of growth. Among them, the fluorescence dilution technique by Helaine and co-workers (Helaine et al., 2010) or the TIMER^bac^ by Bumann and colleagues (Claudi et al., 2014), both established in *Salmonella enterica*, display the great advantage of (i) being transposable to other pathogens as they rely on the differential dilution of fluorescent proteins depending on the division rate (Kerkez et al., 2021; Patel et al., 2021; Personnic et al., 2019; Personnic et al., 2021; Peyrusson et al., 2020) and (ii) being compatible with other fluorescent reporters for virulence (Personnic et al., 2019; Personnic et al., 2021), intracellular niches (Luk et al., 2021) or cues (Cunrath and Bumann, 2019; Schulte et al., 2021). Based on the relationship between the growth rate and ribosome production, Manina et al., constructed a fluorescent reporter strain of *M. tuberculosis* by inserting the gene encoding a destabilized green fluorescent protein at the ribosomal RNA locus. Thereby they identified cryptic subpopulations of nongrowing but metabolically active individuals before and after isoniazid treatment (Manina et al., 2015). Other “persister” reporters include the QUEEN, a single-wavelength sensor to cytosolic ATP (Manuse et al., 2021) or the tetracycline-responsive fluorescent reporter based on the *tet* operon that enables detection of the diffusion of tetracycline derivatives (Raneses et al., 2020). Although robust, lack of growth is not an absolute persisters’ hallmark. Ratiometric fluorescence biosensor of the redox potential of the major mycobacterial antioxidant mycothiol (E_MHS_) revealed that within-host *Mycobacterium* persisters encompass a fraction of the actively replicating bacterial subpopulation featured with distinct redox physiology (Bhaskar et al., 2014; Mishra et al., 2019).

Recent omics profiling (that we termed persistome) unveiled the depth of the differentiation process that render intravacuolar individuals less susceptible to antibiotics (Claudi et al., 2014; Helaine et al., 2014; Mishra et al., 2019; Personnic et al., 2019; Peyrusson et al., 2020; Saliba et al., 2016). In the case of *L. pneumophila*, one can infer from the persister molecular reprogramming that they face and respond to a broad spectrum of stress. it includes: nutritional challenges (e.g. upregulation of the bacterial stringent response; growth arrest; redirected metabolism), host-derived antimicrobials (e.g. upregulation of efflux pumps), oxidative stress (e.g. upregulation of detoxification/repair enzymes). Such features likely contribute to the documented ability of *L. pneumophila* persisters to survive the bactericidal activities of various antibiotics with distinct mode of action, *i.e.*, β-lactams, fluoroquinolones, aminoglycosides and macrolides (Personnic et al., 2019). The transcriptional landscape of *M. tuberculosis* persisters suggests that they experience phagosomal acidification, metal toxicity and reactive oxygen species (ROS). The persister specific physiological realignment involves genes that are known to promote drug refractoriness (*e.g.*, drug efflux pumps such as *mmr*, Rv1258c, and Rv1250) (Mishra et al., 2019). The *S. aureus* persistome also strongly intertwines antibiotic recalcitrance and activation of multiple protective mechanisms triggered within the phagosome (Peyrusson et al., 2020). In addition to the stringent response, the activation of the cell wall stress stimulon (CWSS), a protective response to cell wall defects likely mediate the tolerance to β-lactams. Induction of DNA damage repair system probably contribute to the tolerance to fluoroquinolones while the upregulation of the heat shock stimulon, dealing with damaged proteins, may participate to a higher tolerance of the persisters to both β-lactams and aminoglycosides and possibly influences the action of macrolides. Of note, this persistome was generated under the pressure of the antibiotic oxacillin that could contribute to non-host-derived stress response (Peyrusson et al., 2020).

In addition to those “persister molecular atlas”, the link between host-derived stress and antibiotic persister formation has been deciphered in greater detailed for the acid and oxidative stress.

The phagosomal acidification both efficiently compromises the survival of many microorganisms and contributes to the formation of persisters. In macrophages infected by *S. enterica* Typhimurium, shortly after uptake, drop of the phagosomal pH heterogeneously triggers the bacterial stringent response and the activation of 14 toxin-antitoxin modules. Among them, TacT, is an acetyltransferase that blocks the primary amine group of amino acids on charged tRNA molecules, thereby inhibiting translation and blocking the bacterial division. Growth arrest leads to the inactivation of antibiotic targets and to the observed tolerance to the β-lactam antibiotic cefotaxime (Cheverton et al., 2016; Helaine et al., 2014). In macrophages infected by *M. tuberculosis*, vacuolar acidification alters the redox potential of the major mycobacterial antioxidant mycothiol (MSH), a functional analog to glutathione. This leads to the emergence of a subpopulation of bacteria with reduced MSH. Various cysteine utilization pathways contribute to the biosynthesis of mycothiol and its redox diversity. In the reduced individuals, remodelling of the sulfur metabolism leads to the biogenesis of known protective agents such as H_2_S, low molecular–weight thiols, and iron-sulfur (Fe-S) clusters. In addition, these individuals upregulate various drug efflux pumps. Hence, reprogramming of the redox metabolism in response to host environment increases tolerance to the first line antibiotic isoniazid (Mishra et al., 2019).

The oxidative burst efficiently controls the infection and is mediated by a myriad of reactive oxygen and nitrogen species (ROS/RNS) such as the superoxide anions and nitric oxide that are synthesized by the cellular NADPH oxidase complex and inducible nitric oxide synthase, respectively. Superoxide anions and nitric oxide react to form the potent reactive species peroxynitrite. Once produced, the peroxynitrite can oxidize and nitrate various biological components, including nucleic acids, proteins, and lipids. In macrophages infected by *Staphylococcus aureus*, the peroxynitrite generated during the oxidative burst inactivates the aconitase, an iron-sulfur (Fe-S) cluster-containing enzyme of the tricarboxylic acid (TCA) cycle known to be extremely sensitive to oxidative stress. Collapse of the bacterial TCA cycle reduces the production of ATP, and ultimately, the bacterial entrance into a viable but low metabolic state incompatible with the killing activity of the antibiotic rifampicin (Beam et al., 2021; Rowe et al., 2020). The role of the oxidative burst in the formation of persisters applies to different pathogens. For instance, its induction, upon macrophage stimulation by the pro-inflammatory cytokines IFN-γ, leads to a higher proportion of intracellular *L. pneumophila* persisters (Personnic et al., 2019). The oxidative stress also supports the formation of *M. tuberculosis* persisters (Manina et al., 2015; Saito et al., 2021). Of note, some antibiotics such as clofazimine induces redox-related physiological alterations, *via* an NADH-dependent redox cycling pathway, that contribute to their bactericidal activities, in broth (Dwyer et al., 2014; Grant et al., 2012). Stimulating ROS production was then thought to provide a potential strategy to managing persistent mycobacterial infections. Yet, in the context of an infection, when persisters actually emerge from and respond to oxidative stress, such strategy may reveal counter-productive.

## Persisters survival: undermining the host defense mechanisms

Most pathogens have evolved various strategies to counter host defenses to ensure successful infections and the persisters are no exception. It spans strict stress responses (defensive response, **Figure 1A**) as well as the fine-tuned hijacking of host cell functions (offensive response, **Figure 1B**).

Evading the host bactericidal activities comes at high energy cost. This challenges the established conception according to which persisters are strictly dormant. In agreement, the persistomes mentionned beforehand indicate that the persisters redirect their metabolism, rather than switching it off. Thereby, the intravacuolar persisters can both sustain functional bacterial maintenance and tackle the nutriprive mechanisms at work in the phagosomes. For example, the *L. pneumophila* persisters adopt a fatty acid-based metabolism (Personnic et al., 2019), the *S. aureus* persisters upregulate the galactose metabolism (Peyrusson et al., 2020) and the *S. enterica* Thyphimurium persisters activate the purine and histidine biosynthesis (Claudi et al., 2014).

The persisters defensive response to the host encompasses various mechanisms. *S. aureus* persisters undergo a massive induction of the heat shock stimulon, a central response in stress tolerance crucial to the bacterial protein folding machinery and participating in the degradation of defective proteins (Peyrusson et al., 2020). *S. enterica* Thyphimurium persisters deals with misfolded or damaged proteins in the periplasm by upregulating HtrA, a multifunctional protein quality control factor (Schulte et al., 2021). Intracellular persisters cope with the respiratory burst by deploying anti-oxidant/detoxification responses. *S. enterica* Thyphimurium upregulates the thioredoxin TrxA and the methionine sulfoxide reductase MsrA (Schulte et al., 2021). *L. pneumophila* also produces the thioredoxin TrxA, in addition to the Alkyl hydroperoxide reductase AhpD, the catalase-peroxydase 1 KatG1 and the peroxynitrite reductase (Personnic et al., 2019). Host-derived ROS and RNS induce double DNA breaks (DSBs). *S. enterica* Thyphimurium, *S. aureus*, or *E. coli* persisters actively elicit a program to preserve the genome integrity through the SOS response that contribute to the resilience of persisters to exogenous DNA damaging agents (Demarre et al., 2019; Hill et al., 2021; Peyrusson et al., 2020; Peyrusson et al., 2022).

In addition to the defensive mode, the intracellular *L. pneumophila* persisters execute a specific offensive virulence program that involves a portfolio of only a few tens of effectors (Personnic et al., 2019), out of 400 substrates (Burstein et al., 2016; Finsel and Hilbi, 2015; Personnic et al., 2016), that are translocated into the host cell cytosol *via* the Icm/Dot type 4 secretion system (T4SS) in order to disable host functions. Notably, the persister produce SidC that uses an N-terminal E3 Ub ligase domain to mono-ubiquinate the cellular GTPase Rab1 and mono-ubiquinate and poly-ubiquitinate the GTPase Rab10 (Lockwood et al., 2022). Thereby, the persisters evade the bactericidal phago-lysosomal pathway by redirecting the vacuolar maturation route toward the safer secretory pathway (**Figure 1B**). In agreement, *L. pneumophila* persisters containing vacuole (PCV) exhibits features of the secretory compartments such as the enrichment in calnexin, an endoplasmic reticulum protein, as well as in the phosphoinositide phosphatidylinositol-4-phosphate that is prevalent in the membrane of the Golgi apparatus. In this protective niche, the persisters display higher survival rate and growth resumption capacity (Personnic et al., 2019).

The inability of macrophages to clear the persisters also lies in their discrete bacterial activities, even under antibiotic exposure, that dampens the host response to the infection. The macrophages hosting *Salmonella* persisters deploy an altered pro-inflammatory immune program with profile between the pro-inflammatory M1 and anti-inflammatory M2 polarization states (Saliba et al., 2016; Stapels et al., 2018). To undermine the host cell immune response, the persisters adopt a stealth mode. They exploit the *Salmonella* type 3 secretion system (T3SS) SPI-2 that translocates approximately 30 effectors, some of which are known to downregulate pro-inflammatory responses (Stapels et al., 2018). Notably, the T3SS effector SteE forces the sustained activation of STAT3, a master transcriptional regulator that redirects the macrophage polarization toward a more permissive, anti-inflammatory (M2) phenotype (Gibbs et al., 2020; Panagi et al., 2020; Stapels et al., 2018) (**Figure 1B**). Interestingly, MavC is a newly described T4SS effectors (Gan et al., 2019) specifically produced by the *Legionella* persisters (Personnic et al., 2019). MavC is a transglutaminase that catalyzes the mono-ubiquitination of the E2 enzyme UBE2N. This abolishes UBE2N E2 activity in forming K63-type polyubiquitin chains and dampens the activation of the Nuclear factor-κB (NF-κB) (Gan et al., 2019; Gan et al., 2020; Puvar et al., 2020) (**Figure 1B**), a key regulator of the macrophage M1 polarization (Liu et al., 2017). Perhaps, *Legionella* persisters, similarly to those produced by *S. enterica* Thyphimurium, can control the polarization of the hosting macrophages in order to alleviate the bactericidal pressure.

Surveillance of the cytosol by host pathogen recognition receptors such as the nucleotide oligomerization domain (NOD)-like receptors, is essential to activate the innate immune response and pathogen clearance. Because it confers resistance to intracellular pathogens, many bacterial species have evolved evading strategies. Whether or how persisters deal with the cytosolic sensors that recognize their molecular patterns remain unclear. In non-permissive fibroblasts, the autophagy forces *Salmonella* into growth-arrest, a hallmark of the persisters, in which the peptidoglycan (PG) modifier EcgA is upregulated (Hernandez et al., 2022). The PG of those non-proliferative bacteria undergoes three levels of modifications including atypical crosslinked muropeptides that may contribute to withstanding envelope damage in the harsh phagosomal lumen and atypical muropeptides containing alaninol, which could contribute directly to attenuate immune recognition and the NF-κB pro-inflammatory cascade (Hernandez et al., 2022) (**Figure 1B**). Importantly this PG editing differs from structural alterations known to promote evasion of innate defenses or antibiotic resistance in other pathogens. It is thus tempting to speculate that persister-specific modification of the envelope properties may represent another strategy to evade host defenses.

## Intracellular persisters: heterogeneity on both sides

How individual intracellular bacteria supposedly subjected to similar pressures, embark on different fates remains unclear but likely proceed from the inherent heterogeneity in the biological processes (**Figure 1C**).

At the bacterial level, it has been found that *L. pneumophila* has a bistable activation of the quorum sensing (QS) among an overall homogeneous population of infectious bacteria (Personnic et al., 2021). As the QS fine-tunes the pathogen stress response, it is reasonable to think that individuals may deploy alternative response to host-derived stress and cues depending on their initial priming by the QS. In this regard, inactivation of the *Legionella* QS leads to a drop in within-host persister formation (Personnic et al., 2019). For *S. aureus*, the depth of dormancy seems directly directed by the level of oxidative stress experienced by each individual (Peyrusson et al., 2022). Persisters dormancy would therefore be a dynamic continuum with two extremes that are both antibiotic tolerant: “shallow” persisters that easily escape from dormancy and viable but non-cultivable (VBNC, **Box 1**) cells that are too dormant to resume growth in conventional media (Peyrusson et al., 2022). A similar continuum may explain how seemingly homogeneous intracellular non-replicating *Salmonella*, exhibit extensive bacterium-to-bacterium variations regarding the degree of metabolic activity and regrowth capability (Helaine et al., 2014). It could also determine observed mycobacterial persisters with alternative degree of dormancy (Manina et al., 2015; Saito et al., 2021).

At the subcellular level, following uptake by macrophages, *Mycobacteria* reside in phagosomes with distinct features (e.g. pH) and offering alternative microenvironments that generate as many pathogen subpopulations with a gradient MSH redox potentials (Bhaskar et al., 2014; Manina et al., 2015; Mishra et al., 2019), that, together with alternative antibiotic penetration (Santucci et al., 2021), contribute to the treatment failures. Increased cell-to-cell variation in *Salmonella* purine auxotroph mutant showing a higher proportion of nongrowing cells as compared to the parental strain suggests the coexistence of phagosome with alternative purine availability, resulting in either replication or growth arrest and entry into persistence (Claudi et al., 2014). Across most of the species, the persisters are formed within minutes following phagocytic uptake and the activation of the stringent response. Using the *E. coli* strain LF82 associated with Crohn’s disease, Espeli and coworkers further showed *de novo* persister formation during the intravacuolar exponential expansion that is mediated by the SOS response (Demarre et al., 2019). This suggests that intravacuolar bacterial communities (IBC) would be differentially exposed or would displayed alternative response to host-derived stressors. It is interesting to note that, during the infection, *L. pneumophila* also robustly replicates to eventually forms functionally and spatially structured IBC that heterogeneously responds to ofloxacin exposure (Striednig et al., 2021). One can speculate that, as for *E. coli* LF82, *L. pneumophila* would produce persisters in successive waves through heterogeneous exposure or response to different cues/stressors.

At the host cell level, heterogeneous expression of immune genes leads to the co-existence of macrophages with distinct functional program (M1 and M2 polarization state) (Pham et al., 2020; Stapels et al., 2018). This provides seemingly identical niches with high (M1 pro-inflammatory) or low (M2 anti-inflammatory) bactericidal pressure that may lead to the emergence of *Salmonella* subpopulations and among them the antibiotic persisters (Stapels et al., 2018). In addition, macrophage with different polarization states and bactericidal pressures could determine the observed heterogeneity among persisters by controling the depth of their dormancy, as discussed above.

All this hereabove mentioned heterogeneity is further enhanced in healthy tissues as well as in pathological lesions that displays structured micro-environments and alternative spatio-temporal inflammation dynamics (Bumann and Cunrath, 2017; Burton et al., 2014; Claudi et al., 2014; Davis et al., 2015; Huemer et al., 2021; Manina et al., 2015; Pham et al., 2020).This favors disparate simultaneous encounters. This is especially well illustrated in the mouse model for the typhoid fever. During systemic infection, *Salmonella* targets in priority the spleen but it colonizes this highly structured organ unevenly. The red pulp hosts the majority of the invading *Salmonella* while the near-by spleen white pulp is poorly colonized. In the red pulp, the pathogen is efficiently killed by infiltrating neutrophils and monocytes but survive in resident macrophages. In these host cells, as mentioned before, the pathogen produces antibiotic persisters that survive exposure to the fluoroquinolone enrofloxacin better (Claudi et al., 2014; Li et al., 2021). In the white pulp, treatment with enrofloxacin also fails to clear *Salmonella*. Yet, in this compartment, the treatment outcome is not fully explained by the common “persisters features”, *e.g.*, alternative antibiotic penetration, host stress-induced drug tolerance or pathogen immunomodulatory capabilities. Actually, inflammatory mediators are heterogeneously distributed across splenic compartments, according to the bacterial load, and cooperate with the antibiotic to clear the infection. In the white pulp, the initially low and quickly declining number of neutrophils and monocytes hardly support the bacterial clearance. As *Salmonella* number reaches immune detection limits, inflammatory cells are no-longer recruited and the bacteria largely evade the synergistic killing mediated by both the antibiotic and the immune system (Li et al., 2021).

## Conclusion

Recent research has uncovered various aspects of the peculiar persister biology. The interplay between the pathogen and the host as well as the inherent heterogeneity of the biological processes govern the formation intracellular persisters that divert and get away with both the host-derived and antibiotic-mediated bactericidal activities. Studying the intimate relationships between the persister and the host should provide better understanding of the infection process and innovative strategies to tackle the ongoing antibiotic resistance crisis.

## Author contributions

NP wrote the first draft of the manuscript. PD and SJ wrote sections of the manuscript. All authors contributed to the article and approved the submitted version.

## Glossary

**Table:**

**AST**:	Antimicrobial Susceptibility Testing. A procedure carried out routinely in diagnostic labs on cultivated patient isolates to profile the susceptibility or resistance of a pathogen to a spectrum of anitbiotics (2019).
**Efflux pumps**:	Proteins that are localized in the bacterial envelop and whose function is (i) to recognize noxious agents that have penetrated the organism (or produced by the organism itself) and (ii) to extrude them before they intoxicate the cell (Du et al., 2018).
**Macrophage polarization**:	Process whereby macrophages phenotypically mount a specific phenotype and functional response to different pathophysiological conditions and surrounding microenvironments (Lawrence and Natoli, 2011).
**NF-κB**:	Nuclear factor-κB (NF-κB) represents a family of inducible transcription factors, which regulates a large array of genes involved in different processes of the immune and inflammatory responses. Following activation, NF-κB is rapidly and transiently translocated to the nucleus, mediating induction of various pro-inflammatory genes in innate immune cells (Liu et al., 2017).
**Oxydative burst**:	Also termed respiratory burst is the rapid release of oxidative species, produced by multicomponent enzyme complex in the plasma membrane and phagosomal membrane, to degrade internalized pathogens (Slauch, 2011).
**Pathogen recognition receptors**:	Also termed Pattern Recognition Receptors (PRRs) are proteins capable of recognizing molecules frequently found in pathogens (the so-called Pathogen-Associated Molecular Patterns—PAMPs), or molecules released by damaged cells (the Damage-Associated Molecular Patterns—DAMPs). They are part of innate immune system (Li and Wu, 2021).
**Persistome**:	Persister specific global molecular reprogramming that relies on sustained biosynthetic activities and specific regulatory circuits (Claudi et al., 2014; Helaine et al., 2014; Huemer et al., 2021; Mishra et al., 2019; Personnic et al., 2019; Peyrusson et al., 2020; Saliba et al., 2016).
**Quorum sensing**:	Inter-bacterial communication system based on cell density (Mukherjee and Bassler, 2019).
**SOS response**:	Global response to DNA damage in which the cell cycle is arrested and DNA repair and mutagenesis are induced. The SOS response is under the control of the LexA transcriptional repressor. The LexA regulon includes recombination and repair genes such as *recA* (Maslowska et al., 2019).
**Stringent response**:	The stringent response is a stress signaling system mediated by the alarmone guanosine pentaphosphate [(pp)pGpp] in response to various stress. Broader range of cellular target pathways are controlled by (pp)pGpp, including DNA replication, transcription, nucleotide synthesis, ribosome biogenesis and function, and lipid metabolism (Irving et al., 2021).
**TA modules**:	TA modules are composed of two elements: a stable toxin that poisons the cell by interfering with an essential process such as translation, for example, and an unstable antitoxin that neutralizes its cognate toxin (Singh et al., 2021).
**VBNC**:	Viable but non-cultivable bacteria have been defined as cells which, induced by some stress, become nonculturable on media that would normally support their growth (Ayrapetyan et al., 2018).
